# Lithium chloride enhances serotonin induced calcium activity in EGFP-GnIH neurons

**DOI:** 10.1038/s41598-020-70710-x

**Published:** 2020-08-17

**Authors:** Chuin Hau Teo, Tomoko Soga, Ishwar Parhar

**Affiliations:** Brain Research Institute, Jeffery Cheah School of Medicine and Health Sciences, Monash University Malaysia, 47500 Bandar Sunway, Selangor Malaysia

**Keywords:** Cell biology, Neuroscience

## Abstract

Neurons synthesizing gonadotropin-inhibitory hormone (GnIH) have been implicated in the control of reproduction, food intake and stress. Serotonin (5-HT) receptors have been shown in GnIH neurons; however, their functional role in the regulation of GnIH neurons remains to be elucidated. In this study, we measured intracellular calcium ion levels following 5-HT treatment to hypothalamic primary cultures of enhanced fluorescent green protein-tagged GnIH (EGFP-GnIH) neurons from Wistar rat pups of mixed sex. Three days after initial seeding of the primary cultures, the test groups were pre-treated with lithium chloride to selectively inhibit glycogen synthase kinase 3 beta to promote intracellular calcium levels, whereas the control groups received culture medium with no lithium chloride treatment. 24 h later, the cultures were incubated with rhodamine-2AM (rhod-2AM) calcium indicator dye for one hour prior to imaging. 5-HT was added to the culture dishes 5 min after commencement of imaging. Analysis of intracellular calcium levels in EGFP-GnIH neurons showed that pre-treatment with lithium chloride before 5-HT treatment resulted in significant increase in intracellular calcium levels, two times higher than the baseline. This suggests that lithium chloride enhances the responsiveness of GnIH neurons to 5-HT.

## Introduction

Gonadotropin-inhibitory hormone (GnIH) was discovered in birds as an antagonist to gonadotropin-releasing hormone (GnRH)^1^. Since then, GnIH has been identified in many mammalian and non-mammalian vertebrates including rodents, bovines and primates^2–5^. The function of GnIH has been highly conserved across vertebrates, acting to inhibit GnRH-mediated gonadotrophin release with some exceptions found in teleosts^1,4–8^. Besides its role in reproduction and appetite^9^, GnIH has been implicated in depressive-like behaviour and stress^10,11^.

A number of hormonal factors (melatonin, glucocorticoids, estrogen and thyroid hormones)^12,13^ have been suggested to regulate GnIH neurons. However, the regulation of GnIH neurons by neurotransmitters is not well-identified, except for serotonin (5-HT). GnIH neurons co-express 5-HT receptors (5-HT1A, 1B, 1D, 1F, 2A, 2B, 3A, 5A, 5B, as well as 5-HT6 and 5-HT7)^14^; the selective serotonin reuptake inhibitor, citalopram (anti-depressant) increases GnIH neuronal numbers in the dorsomedial hypothalamus^14^. Social stress decreases 5-HT fiber innervations to GnIH neurons^15^ but increases the expression levels of beta-catenin within GnIH neurons, suggesting increased activity of the Wnt signalling pathway, which can result in transcriptional activation or repression of cellular machinery^16^. Impairment of 5-HT function under social stress^17^ indicates a potential connection between 5-HT regulation and beta-catenin expression. Indeed, 5-HT (5-HT1A, 5-HT1D, 5-HT2, 5-HT7) receptors are known to modulate beta-catenin activity^18–21^ and fluoxetine, a selective serotonin reuptake inhibitor, increases nuclear localization of beta-catenin^22^. Translocation of beta-catenin into the nucleus is facilitated by GSK3β, which is part of the Wnt signaling pathway^23,24^; this promotes an increase in intracellular calcium^25^.

The 5-HT and the GnIH system in the brain have a strong association with stress and reproduction^26^, but how these two neuronal systems interact with each other is less known. Despite the presence of 5-HT receptors in GnIH neurons^14^, the effect of 5-HT on the internal dynamics of GnIH neurons remains to be investigated, which we hypothesize is through the GSK3β-Wnt signaling pathway. Therefore, the objective of this study was to treat rat EGFP-GnIH neurons in hypothalamic primary cultures with 5-HT and selectively inhibit GSK3β using lithium chloride (LiCl) to observe change in the activity of EGFP-GnIH neurons using calcium imaging. Calcium imaging measures the intensity of fluorescent dyes bound to free calcium ions in the cytosol^27^. This allows microscopic imaging of activity within the cell as an alternative to electrophysiology and patch clamp methods. Thus, the elucidation of GSK3β-Wnt signaling pathway, by which 5-HT regulates GnIH, will provide a new avenue for future studies to explore the complexity of stress-induced reproductive dysfunction.

## Results

### Live cell calcium imaging

**Figure 1:**
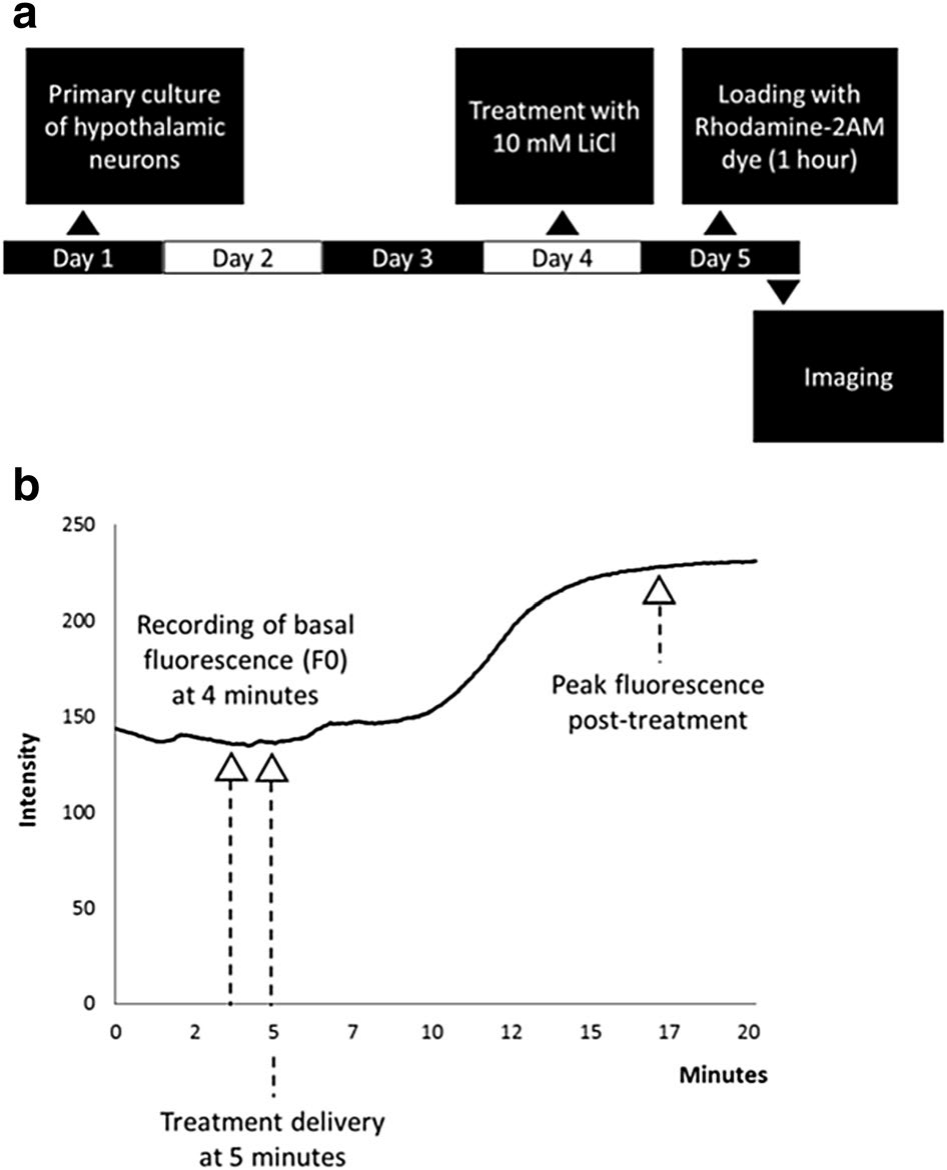
Flow-chart for primary cell culture and imaging process. (**a**) The flowchart depicts the process in the primary culture of hypothalamic neurons. (**b**) The steps involved in imaging the neurons.

Neurons were observed at day 5 of the primary culture, with treatment being performed 1 day prior (Fig. 1). During imaging, basal fluorescence (F_0_) was determined at 4 min after commencement of imaging. 5-HT treatment was delivered at 5 min and peak fluorescence measured at any time post treatment.

**Figure 2:**
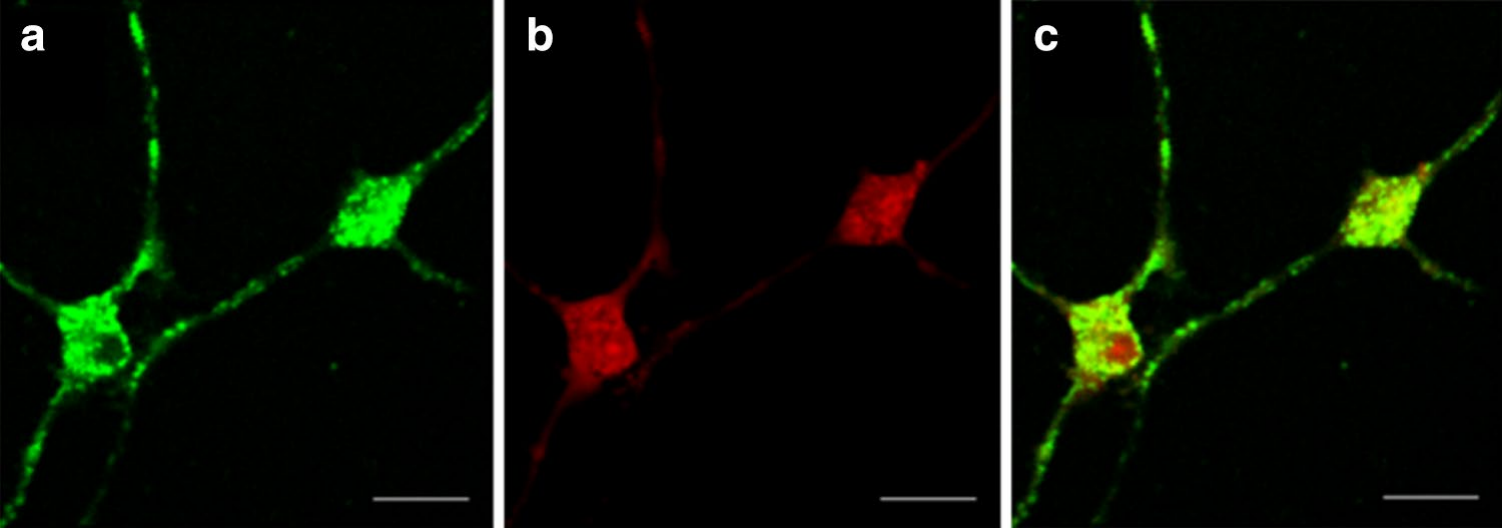
Loading of neurons with Rhod-2AM at differing concentrations. (**a**) EGFP-GnIH fluorescent neuron (green), (**b**) Rhodamine-2AM (rhod-2AM) calcium indicator staining (red), (**c**) colocalization of rhod-2AM within EGFP-GnIH neurons. Scale bar = 25 µm.

Cultured GnIH neurons (Fig. 2a) (defined as neurons with a measured green fluorescent intensity of > 50 during imaging), were successfully loaded with rhod-2AM (Fig. 2b) at 5 µM. Imaging of both dyes showed co-localization of the dye with GnIH neurons (Fig. 2c).

**Figure 3:**
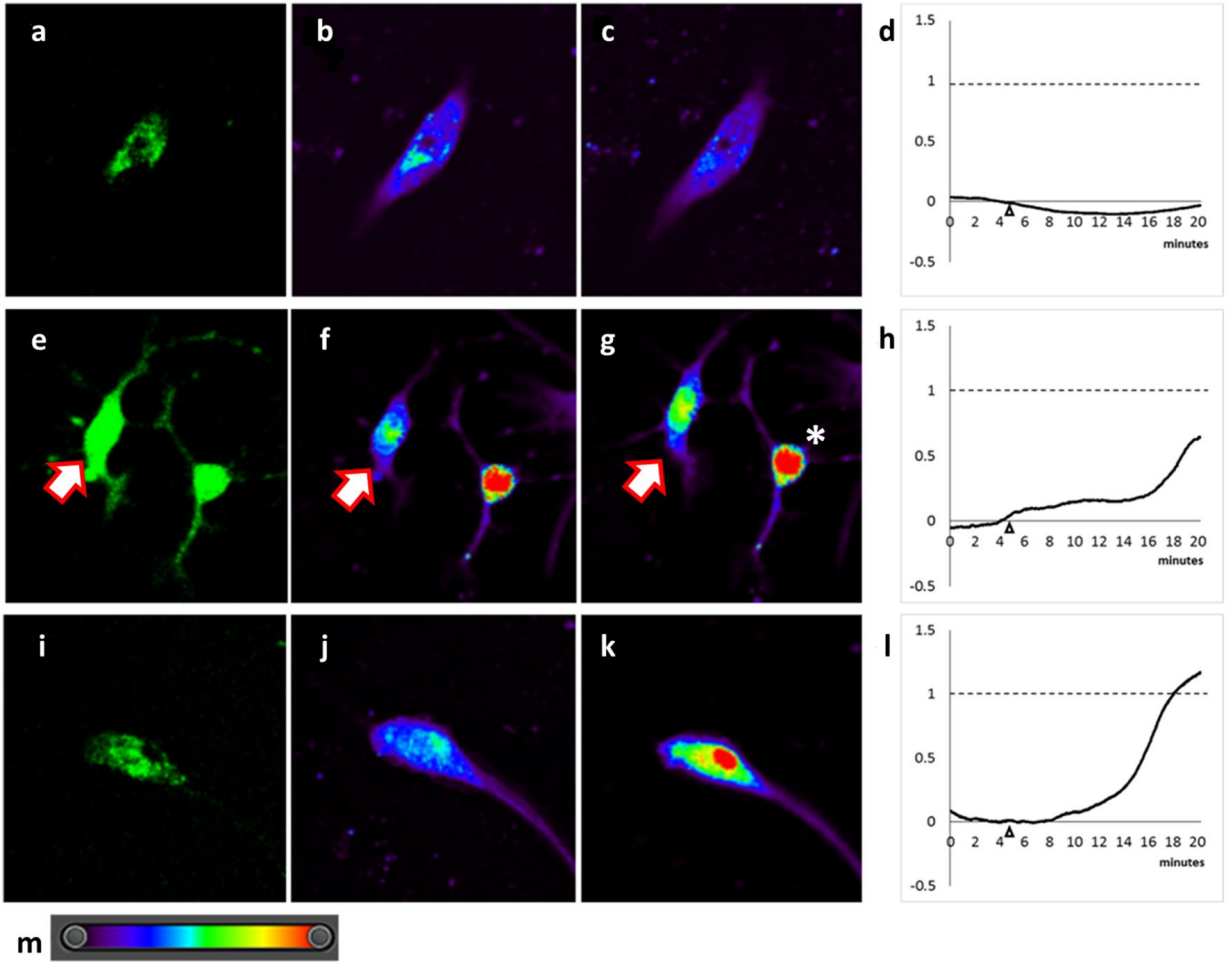
Calcium imaging of day-5 primary culture GnIH neurons from the dorsomedial hypothalamus. (**a**) Typical GnIH neuron incubated with LiCl (**b**) before treatment and (**c**) 10 min after placebo treatment (H_2_O). (**d**) Example of relative change intensity in neurons treated with placebo. (**e**) Typical GnIH neuron not incubated with LiCl (**f**) before treatment and (**g**) 10 min after 5-HT treatment. (**h**) Example of relative change intensity in neurons treated with 5-HT only. (**i**) Typical GnIH neuron incubated with LiCl (**j**) before treatment and (**k**) 10 min after 5-HT treatment. (**l**) Example of relative change intensity in neurons pre-treated with LiCl and treated with 5-HT. (**m**) Spectrum for the pseudo-colour representation used to represent calcium intensity. Blue indicates low and red indicates high calcium intensity. White arrow in images **e**–**g** indicates GnIH neuron used for measurement in **h**. *Shows a cell with high rhod-2AM fluorescent intensity before treatment (unused in 5-HT response calculations). Scale bar = 25 µm.

The change in fluorescent intensity was measured by calculating the change in intensity, which is defined as the difference between the peak fluorescence post-treatment and the basal fluorescence. This can be represented by the term ΔF. Treatment of GnIH neurons with water as a placebo failed to elicit a significant response (Fig. 3a–c). The change in intensity of the rhod-2AM dye in those neurons remained low (Fig. 3d). In GnIH neurons not incubated with LiCl but treated with 5-HT (Fig. 3e–g), a small response was also observed (Fig. 3h). GnIH neurons pre-treated with LiCl and treated with 5-HT tend to demonstrate an increase in intensity after the 5-HT treatment (Fig. 3i–k), with a hike in intensity (Fig. 3l) observed shortly after treatment.

**Figure 4:**
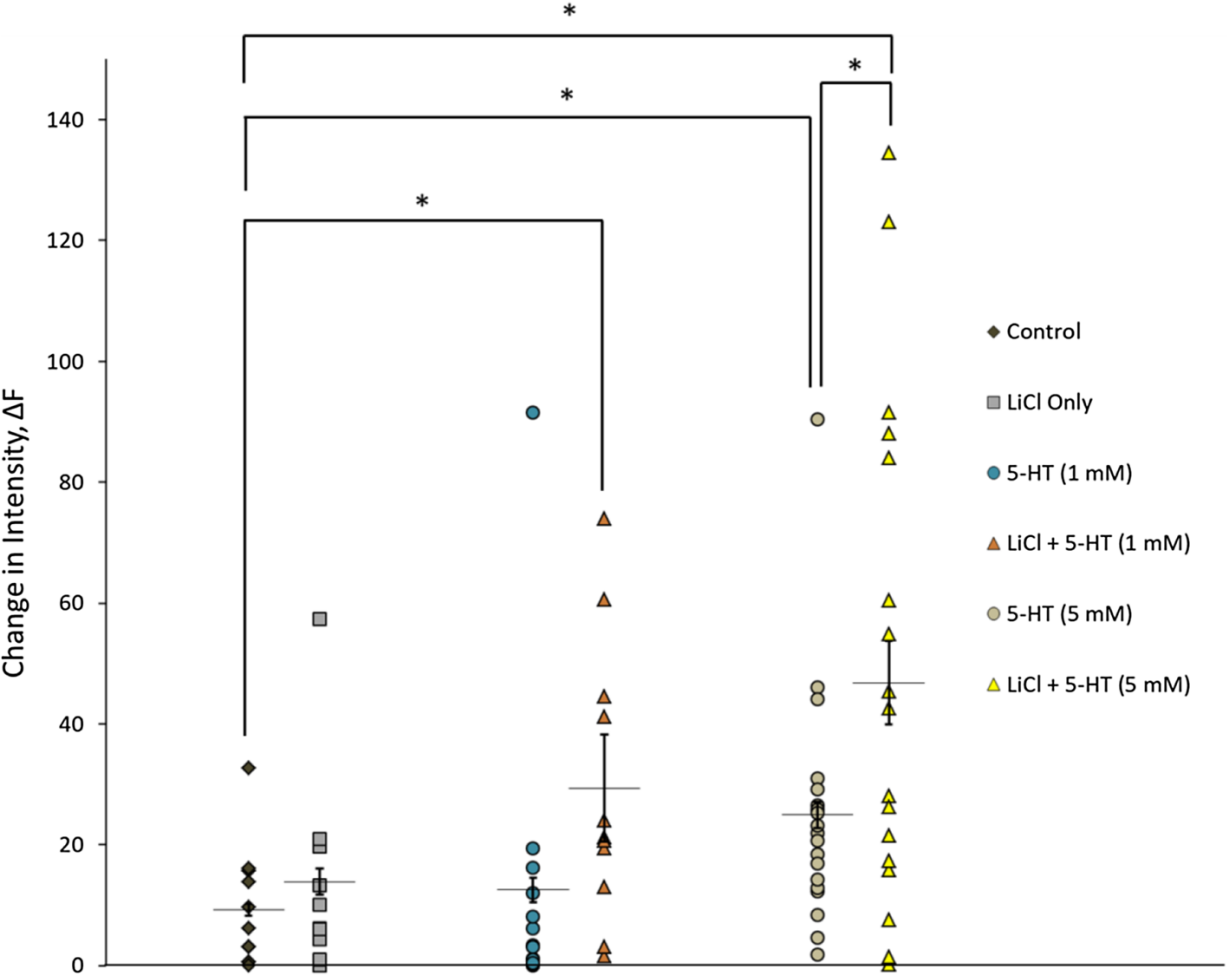
Effect of LiCl and 5HT treatment on calcium levels in GnIH neuron primary culture. Measurement of change in fluorescent intensity of each neuron charted as a scatterplot of relative change in intensity under LiCl pre-treatment with differing doses of 5-HT concentration. The treatments consist of control (average ΔF = 9.247, n = 11), LiCl only (average ΔF = 13.88, n = 10), 5-HT (1 mM) (average ΔF = 12.53, n = 13), LiCl + 5-HT (1 mM) (average ΔF = 29.38, n = 11), 5-HT (5 mM) (average ΔF = 24.94, n = 19), LiCl + 5-HT (5 mM) (average ΔF = 46.82, n = 18). Data in scatterplot is presented as means ± SEM for ΔF. Significance was set at *p < 0.05.

A total of 82 GnIH neurons were observed across five independent experiments (Fig. 4). The ΔF for all GnIH neurons counted in their respective populations were averaged together and grouped by treatment. One neuron exhibited a marked increase in fluorescent intensity in the control group after the placebo treatment. Of the neurons pre-treated with only LiCl, a single neuron exhibited a notably positive change in fluorescent intensity, with the average intensity not significantly different from the control. GnIH neurons treated with 5 mM 5-HT exhibited significant differences from the control on average, although the same was not observed when comparing 1 mM 5-HT treatment with the control (5-HT (5 mM): 24.94 ± 2.163 [n = 19] and control: 9.247 ± 0.9391 [n = 11], *p* < 0.05, Fig. 4). The 5 mM 5-HT treatment yielded a higher change in fluorescent intensity than 1 mM 5-HT on average, but the difference between the two was not found to be statistically significant.

Neurons pre-treated with LiCl followed by treatment with 5 mM 5-HT showed a significantly greater increase in fluorescent intensity compared to treatment with 5 mM 5-HT alone and the control group, suggesting heightened calcium activity. (LiCl + 5-HT (5 mM): 46.82 ± 6.867 [n = 18] and 5-HT (5 mM) 24.94 ± 2.163 [ n = 19], *p* < 0.05; LiCl + 5-HT (5 mM): 46.82 ± 6.867 [n = 18] and control: 9.247 ± 0.9391 [n = 11], *p* < 0.05; Fig. 4). Furthermore, a significant difference was also observed with neurons pre-treated with LiCl and 5-HT 1 mM treatment when compared to the control (LiCl + 5-HT (1 mM): 29.38 ± 8.859 [n = 11] and control: 9.247 ± 0.9391 [n = 11], *p* < 0.05; Fig. 4).

## Discussion

Primary cultures treated with 5-HT alone or in combination with LiCl showed an increased percentage of activated GnIH neurons and an increase in intracellular calcium levels within those neurons. This shows that 5-HT can regulate GnIH neurons.

In this study, a higher concentration of 5-HT was utilized (5 mM) to elicit a more pronounced response from the neurons. Since a higher concentration of 5-HT has been utilized in primary cell cultures in calcium imaging experiments^28^, it is not likely to be adverse to the cells. GnIH neurons treated with only 5 mM 5-HT demonstrated increased fluorescence intensity, but the responsiveness of GnIH neurons to 5-HT treatment was not significant in the 1 mM in comparison to 5 mM treatment. This suggests that a small scale calcium elevation in GnIH neurons, in response to 5-HT, occurs even in the absence of LiCl treatment. The elevation in calcium was measured in the span of minutes, indicating a slower, global transient pattern involved in internal cellular signalling^29^.

It is possible that the measurement of intracellular calcium levels can be influenced by neuronal potentiation due to interaction between matured neurons after their 14th division^30–32^. However, in the present study, it is unlikely that 5-HT neuronal network activity could have affected our observations because we measured intracellular calcium levels in GnIH neurons in primary cultures that are unlikely to have synaptic transmission. Secondly, our primary cultures were at an immature stage, where the GnIH neurons are approximately at the 4^th^ division and, therefore, could not be influenced by other neurons. However, the effect of 5-HT can differ depending on the developmental stage of the brain^33^, and as such the response of neurons in primary culture to 5-HT may depend on the age at which they are treated.

While lithium treatment has an attenuative effect on intracellular calcium signalling^25,34,35^, here we observed that pre-treatment with LiCl enhances the responsiveness of GnIH neurons to 5-HT treatment, which further increased intracellular calcium levels. In particular, the calcium intensity in the 1 mM 5-HT treatment was bolstered by LiCl to a notable degree as the majority of the neurons in that group were not responsive to 5-HT in the absence of LiCl. Thus, it can be speculated that the selective inhibition of GSK3β by LiCl^36^ could have activated the Wnt canonical pathway and thereby facilitate the translocation of beta-catenin into the nucleus^23,24^, which promoted an increase in intracellular calcium. This is similar to lymphoblast, glioma cell lines^25,37^ and cortical neurons^38^ where lithium treatment increases intracellular calcium levels. Further investigations are needed to elucidate the nature of 5-HT induced Wnt signalling in GnIH neurons. Of particular importance would be the use of thapsigargin, which has been shown to block beta-catenin, part of the GSK3β-Wnt signalling pathway, that induces intracellular calcium release^39^.

In conclusion, this study shows that LiCl treatment enhances intracellular calcium response to 5-HT in GnIH neurons. It can be speculated that this enhancement is due to the inhibition of the GSK3β-Wnt signalling pathway by lithium. This finding will provide a new avenue for future studies to explore the role of 5-HT-GnIH in stress-induced reproductive dysfunction.

## Methods

### Animals and housing conditions (primary culture)

In this experiment, we used 48 EGFP-GnIH Wistar rat pups of mixed sex on post-natal day 5. We created these transgenic rats using rat GnIH promoter (3 kbp upstream from the start codon)-driven EGFP expression plasmid and confirmed the genotyping and phenotype in the brain^10^. A 12 h-long light/dark cycle (lights on from 12:00 am till 12:00 pm) was established for the rats. The room temperature was kept steady at ± 22 °C with humidity maintained at stable levels. This environment and light/dark cycle was continued for the duration of the housing. Food and water was made available ad libitum for the animals. Animal welfare and experimental ethics were in line with the authorized guidelines laid out by the Animal Ethics Committee of Monash University, which is in compliance with the Australian code for the care and use of animals for scientific purposes (2013). An approval code from the committee, AEC (MARP/2017/021 [2017–2020]), was obtained for the primary culture study. All experimental procedures were approved and performed under the guidelines given by the Monash University.

### Primary culture

Extraction of brains from 48 postnatal day 5 EGFP-GnIH rat pups was conducted by first placing the pups in an air-tight polystyrene container with dry ice (solid CO_2_) in order to anesthetize them. The pups were then decapitated with scissors, the skulls opened and the brains harvested to be put into 25 mL of Krebs buffer (119 mM NaCl, 2.5 mM KCl, 1.0 mM NaH_2_PO_4_, 2.5 mM CaCl_2_·2H_2_O, 1.3 mM MgCl_2_·6H_2_O, 20 mM HEPES, 11 mM glucose) at 4 °C. The brains were quickly transferred to a dissection dish as soon as possible. The total number of independent observations in this experiment was five, taken from five batches of pups delivered from separate mothers.

The brains were dissected with Adult Mouse Brain Slicer Matrix with 1.0 mm coronal section slice intervals. The hypothalamus was removed from the brain slices and collected into 5 mL 37 °C Tryple Express (Gibco Tryple Express, Thermofisher Scientific, MA, USA) solution before being incubated for 20 min. The medium was removed slowly and carefully to avoid any bubbling. 5 mL of fresh Krebs buffer was then added and incubation performed at 37 °C for 5 min. The medium was removed once again, fresh 5 mL Krebs buffer added, and incubated at 37 °C for 5 min. The medium was slowly removed before adding fresh 2.5 mL of Krebs buffer and 5 µL of DNase I. The cells were triturated gently and seeded at 500 µL/dish to a coated 35 mm Eppendorf glass-bottomed dish (Eppendorf Cell Imaging Dish 145 µm, Eppendorf AG, Hamburg, Germany). Neurobasal A medium (Neurobasal-A medium minus phenol red, Thermo-Fisher Scientific, MA, USA) was supplemented with 2% serum-free B-2 (17,504,044, Gibco B-27 Supplement 50X, Thermo-Fisher Scientific, MA, USA) 0.5 mM l-glutamine (Gibco l-glutamine, Thermo-Fisher Scientific, MA, USA), 30 mM glucose (d-glucose anhydrous, Fisher Scientific UK, Loughborough, UK) and 14.2 uL/mL 100× antibiotic–antimycotic solution (Gibco Antibiotic–Antimycotic 100×, Thermo-Fisher Scientific, MA, USA). 1 mL of Neurobasal A medium was added to each dish and incubated at 37 °C for 2 h. The dishes were examined to ensure most of the cells have attached. Afterwards, the medium was replaced with 37 °C Neurobasal A medium (2 mL/well) and allowed to incubate at 37 °C for 18 h. Medium change was performed every 2 days using fresh Neurobasal A medium to replace 3/4ths (1.5 mL) of the existing medium.

### Primary culture treatment

Treatment of the primary culture was performed on day 4 of the culture. 200 µL of 100 mM LiCl (Takara Bio, Japan) was added to the culture dish containing 2 mL of Neurobasal A medium for a final concentration of 10 mM LiCl. The dish was allowed to incubate for 24 h before imaging. Dishes assigned for no LiCl pre-treatment were instead treated with 200 µL of ultrapure miliQ H_2_O replacing the LiCl stock solution.

### Calcium dye loading

Calcium dye loading was performed on day 5 of the culture and one hour before imaging. 10 µL of Stock Rhod-2AM calcium dye (1 mg/mL, AB142780, Abcam Inc, MA, USA) in DMSO was mixed with 10 µL of pluronic F-127 (20% DMSO) and vortexed. The resulting solution was pipetted directly on top of the dish in 990 µL Krebs buffer at room temperature (rhod-2AM 5 µM, DMSO v/v < 0.1%). The dish was wrapped in aluminium foil and allowed to incubate for 1 h at 37 °C. The cells were then washed in 37 °C 1 mL Krebs buffer twice for 10 min to remove the excess dye. 37 °C 2 mL Krebs buffer is then added prior to commencing with imaging.

### Imaging

The Leica laser-scanning multiphoton microscope (Leica Microsystems, Germany) was used for measuring activity of GnIH neurons loaded with Rhod-2AM dye. The EGFP and rhod-2AM fluorophores were excited by lasers emitting at wavelengths of 488 nm and 581 nm respectively. Using the Leica Application Suite X (LAS X) software, the dish was observed live for 5 min. 100 µL of 100 mM serotonin creatinine sulfate complex (5-HT) was added to the glass bottom dish for a final concentration of 5 mM 5-HT before proceeding to record for another 15 min. Control dishes that would not undergo 5-HT treatment were instead treated with 100 µL of ultrapure miliQ H_2_O replacing the 5-HT solution. Activity of GnIH neurons (green fluorescence) was determined by the intensity of rhod-2AM dye (red fluorescence) which increases with calcium activity. While a threshold for intracellular calcium levels is normally set for measurement of action potentials^40^, the measurement of internal calcium signalling, particularly of rhodamine-2AM dye is performed without setting thresholds; instead, it is a direct measure of change in fluorescent intensity^41–43^. Dosage response was measured by repeating the experiment with 20 µL of 100 mM 5-HT for a final concentration of 1 mM 5-HT.

### Data analysis and statistics

Basal pre-treatment fluorescence (F_0_) was recorded at 4 min after imaging had commenced using the Leica Application Suite X (LAS X) software. A number of EGFP-GnIH neurons were already highly fluorescent prior to treatment. As such, all EGFP-GnIH neurons exhibiting rhodamine-2AM fluorescence at an initial intensity value of lower than 100 were chosen to observe the 5-HT response in order to omit neurons unsuitable for 5-HT response analysis. Peak fluorescence was selected from the highest fluorescent intensity readings at any time post-treatment. Change in fluorescent intensity (ΔF) was calculated by measuring the difference in intensity between current fluorescence and basal fluorescence. Data is presented as means ± SEM for comparison of relative change, and analysed using a one way ANOVA post hoc test in order to determine whether there were any statistically significant differences between the means of the different treatment categories. Significance was set as *p* < 0.05.

## Data Availability

The datasets generated during and analysed during the current study are available from the corresponding author on reasonable request.
